# Cognitive impairment in post-acute sequelae of COVID-19 and short duration myalgic encephalomyelitis patients is mediated by orthostatic hemodynamic changes

**DOI:** 10.3389/fnins.2023.1203514

**Published:** 2023-06-26

**Authors:** Heather Day, Brayden Yellman, Sarah Hammer, Candace Rond, Jennifer Bell, Saeed Abbaszadeh, Greg Stoddard, Derya Unutmaz, Lucinda Bateman, Suzanne D. Vernon

**Affiliations:** ^1^School of Medicine, The University of Utah, Salt Lake City, UT, United States; ^2^Bateman Horne Center, Salt Lake City, UT, United States; ^3^Jackson Laboratory for Genomic Medicine, School of Medicine, University of Connecticut, Farmington, CT, United States

**Keywords:** post-acute sequelae of SARS-CoV-2 (PASC), long COVID, ME/CFS, myalgic encephalomyelitis, cognitive impairment, orthostatic hemodynamic changes

## Abstract

**Introduction:**

Cognitive impairment is experienced by people with myalgic encephalomyelitis/chronic fatigue syndrome (ME/CFS) and post-acute sequelae of COVID-19 (PASC). Patients report difficulty remembering, concentrating, and making decisions. Our objective was to determine whether orthostatic hemodynamic changes were causally linked to cognitive impairment in these diseases.

**Methods:**

This prospective, observational cohort study enrolled PASC, ME/CFS, and healthy controls. All participants underwent clinical evaluation and assessment that included brief cognitive testing before and after an orthostatic challenge. Cognitive testing measured cognitive efficiency which is defined as the speed and accuracy of subject’s total correct responses per minute. General linear mixed models were used to analyze hemodynamics and cognitive efficiency during the orthostatic challenge. Additionally, mediation analysis was used to determine if hemodynamic instability induced during the orthostatic challenge mediated the relationship between disease status and cognitive impairment.

**Results:**

Of the 276 participants enrolled, 256 were included in this study (34 PASC, 71 < 4 year duration ME/CFS, 69 > 10 year ME/CFS duration, and 82 healthy controls). Compared to healthy controls, the disease cohorts had significantly lower cognitive efficiency scores immediately following the orthostatic challenge. Cognitive efficiency remained low for the >10 year ME/CFS 2 and 7 days after orthostatic challenge. Narrow pulse pressure less than 25% of systolic pressure occurred at 4 and 5 min into the orthostatic challenge for the PASC and ME/CFS cohorts, respectively. Abnormally narrow pulse pressure was associated with slowed information processing in PASC patients compared to healthy controls (−1.5, *p* = 0.04). Furthermore, increased heart rate during the orthostatic challenge was associated with a decreased procedural reaction time in PASC and < 4 year ME/CFS patients who were 40 to 65 years of age.

**Discussion:**

For PASC patients, both their disease state and hemodynamic changes during orthostatic challenge were associated with slower reaction time and decreased response accuracy during cognitive testing. Reduced cognitive efficiency in <4 year ME/CFS patients was associated with higher heart rate in response to orthostatic stress. Hemodynamic changes did not correlate with cognitive impairment for >10 year ME/CFS patients, but cognitive impairment remained. These findings underscore the need for early diagnosis to mitigate direct hemodynamic and other physiological effects on symptoms of cognitive impairment.

## Introduction

Evidence that myalgic encephalomyelitis (ME/CFS) is a prototypical post-infectious disease dates to an epidemic in Iceland in 1949 that simulated poliomyelitis and many had still not recovered 6 years later (Sigurdsson et al., 1950; Sigurdsson, 1956). There is a recognition of the chronic consequences of acute infection, such as chronic arthritis from acute infection with chikungunya virus or long-term, neurological changes from West Nile virus (Baldwin et al., 2016; Pathak et al., 2019; Clé et al., 2020). Now with more than 750 million confirmed cases of COVID-19 worldwide [WHO Coronavirus (COVID-19) dashboard]^1^ research on post-acute sequelae of COVID-19 (PASC) has intensified. It is estimated at least 10% of people that had COVID-19 will continue to experience symptoms for more than 3 months (Centers for Disease Control and Prevention)^2^. Although there are unique signs and symptoms in response to any acute infection, there is also a common, stereotyped post-infectious syndrome characterized by fatigue that impairs physical function, post-exertional malaise, unrefreshing sleep, orthostatic intolerance, and cognitive impairment (Hickie et al., 2006). ME/CFS and many with PASC share these stereotyped post-infectious symptoms.

Dysautonomia occurs when the nerves that regulate nonvoluntary functions, such as heart rate and blood pressure, malfunction (Karemaker, 2017). Orthostatic intolerance (OI) describes the signs and symptoms that occur in upright posture and improve when supine and may include an inability to stay upright due to dizziness and fainting, fast or slow heartbeat, inappropriate drop or rise of blood pressure, and cognitive problems, to name a few (Rowe, 2002). OI is included in the diagnostic criteria for ME/CFS (Institute of Medicine, 2015) and is now widely recognized as one of the primary clinical manifestations of PASC (Jammoul et al., 2023). Cognitive impairment that results in difficulty remembering, concentrating, and making decisions is prevalent in both ME/CFS and PASC (Komaroff and Bateman, 2021; Komaroff and Lipkin, 2021). Recently, we demonstrated that when ME/CFS and PASC patients underwent an orthostatic challenge, cognitive reaction time significantly worsened in both ME/CFS and PASC patients (Vernon et al., 2022).

This led us to hypothesize that orthostatic intolerance and the hemodynamic changes that occur during orthostatic challenge may be causing or contributing to cognitive impairment in both ME/CFS and PASC. To test this, a brief cognitive assessment was done before and immediately after a passive stand test and then 2 and 7 days later. Mediation analysis was used to identify and attempt to explain the extent to which disease status and hemodynamic instability explain impaired cognition.

## Materials and methods

### PASC cohort

Between April and September 2021, we enrolled PASC patients who were 18 to 65 years old with symptoms of fatigue, exercise intolerance or other unwellness that persisted for at least 3 months and that the participant, or their doctor thought were related to COVID-19. Other inclusion criteria were good general health prior to sickness with COVID-19 and evidence of SARS-CoV-2 by PCR or antigen (provided by the subject or their provider) or presence of IgG antibodies to SARS-CoV-2 prior to receiving COVID-19 vaccination. Exclusion criteria included, (1) hospitalized for >72 h for COVID-19, (2) documented organ damage as a result of COVID-19 infection, (3) other active and untreated disease processes that explain the major symptoms of fatigue, sleep disturbance, pain, and cognitive dysfunction, (4) untreated primary sleep disorders, (5) rheumatological disorders, (6) immune disorders, (7) neurological disorders, (8) other infectious disease, (9) psychiatric disorders that alter perception of reality or ability to communicate clearly or impair physical health and function, and (10) laboratory testing or imaging are available that support an alternate exclusionary diagnosis.

### ME/CFS cohorts

Beginning in January 2018, we enrolled ME/CFS patients who had been sick for <4 years or sick for >10 years. No ME/CFS patients with duration ≥4 years and ≤ 10 years were enrolled in order to have clear distinctions between short and long duration of illness with ME/CFS. All participants were 18 to 65 years old at the time of enrollment. ME/CFS diagnosis according to the Institute of Medicine clinical diagnostic criteria and disease duration of <4 years were confirmed during clinical differential diagnosis and thorough medical work up (Institute of Medicine, 2015). Additional inclusion criteria required, (1) a substantial reduction or impairment in the ability to engage in pre-illness levels of occupational, educational, social, or personal activities that persists for more than 6 months and less than 4 years and is accompanied by fatigue, which is often profound, is of new or definite onset (not lifelong), is not the result of ongoing excessive exertion, and is not substantially alleviated by rest, and (2) post-exertional malaise. Exclusionary criteria for the <4 year ME/CFS cohort were, (1) morbid obesity BMI > 40, (2) other active and untreated disease processes that explain most of the major symptoms of fatigue, sleep disturbance, pain, and cognitive dysfunction, (3) untreated primary sleep disorders, (4) rheumatological disorders, (5) immune disorders, (6) neurological disorders, (7) infectious diseases, (8) psychiatric disorders that alter perception of reality or ability to communicate clearly or impair physical health and function, (9) laboratory testing or imaging are available that support an alternate exclusionary diagnosis, and (10) treatment with short-term (less than 2 weeks) antiviral or antibiotic medication within the past 30 days.

For the >10 year ME/CFS cohort, disease duration of >10 year and clinical criteria was confirmed to meet the Institute of Medicine criteria for ME/CFS during clinical evaluation and medical history review (Institute of Medicine, 2015). Other than disease duration, inclusion and exclusion criteria were the same as for <4 year ME/CFS cohort.

### Healthy control cohort

Healthy control participants were also between 18 to 65 years of age and in general good health. Enrollment began in 2018 and subjects were selected to match the <4 year ME/CFS cohort by age (within 5 years), race, and sex (~2:1 female to male ratio). Exclusion criteria for healthy controls included, (1) a diagnosis or history of ME/CFS, (2) morbid obesity BMI > 40, (3) treatment with short-term (less than 2 weeks) antiviral or antibiotic medication within the past 30 days or (4) treatment long-term (longer than 2 weeks) antiviral medication or immunomodulatory medications within the past 6 months.

Approval was received before enrolling any subjects in the study (The Jackson Laboratory Institutional Review Board, 17-JGM-13). All participants were educated about the study prior to enrollment and signed all appropriate informed consent documents. Research staff followed Good Clinical Practices (GCP) guidelines to ensure subject safety and privacy.

### Cognitive testing

Upon arrival at the Bateman Horne Center, participants downloaded the Defense Automated Neurobehavioral Assessment (DANA) Brain Vital app to their smartphones (Lathan et al., 2013). DANA Brain Vital is a test that includes three reaction time and information processing measurements: simple reaction time (SRT), procedural reaction time (PRT), and sustained attention or Go-No-Go (GNG) (Resnick and Lathan, 2016). The test results are reported as cognitive efficiency calculated accuracy x speed x 60,000. SRT is a simple reaction time task in which the user taps an orange target symbol as soon as it appears on the screen. PRT incorporates choice by having the user differentiate between two sets of characters: a 2, 3, 4, or 5 appears on the screen and the user taps one of two buttons [2 or 3] or [4 or 5]. GNG is a forced choice measure of reaction time where either a gray foe or green friend appears on the screen. The user is instructed to tap the screen only when the gray foe appears. Participants completed DANA Brain Vital just before then immediately after the orthostatic challenge and again at home 2 and 7 days after the orthostatic challenge.

### Orthostatic challenge

The 10-min NASA Lean Test (NLT) is a standardized passive stand test and was used as the orthostatic challenge in this study (Lee et al., 2020). Briefly, participants rested supine on an exam table for 10 min. Baseline blood pressure (BP) and heart rate (HR) were measured twice during the last 2 min of the supine rest. Participants then stood with only their shoulder blades touching the wall. Their heels were positioned 6–8 inches from the wall. Systolic BP (SBP), diastolic BP (DBP), and HR were recorded every minute during the 10-min NLT. Pulse pressure (PP) was calculated according to the consensus equation: PP = SBP – DBP. Peripheral perfusion was roughly estimated using PP/SBP, defined as abnormal PP/SBP less than 25% (Homan et al., 2022).

### Data analysis

To estimate the effect between disease status and cognition after the NLT, we ran three different general linear models comparing baseline measurements to the three time points (immediately post-NLT, 2 and 7 days post NLT). A general linear mixed model with time, group, and time x group interaction as fixed effects and subject ID as the random effect, was used to compare the post-fit marginal estimates of the average effect (5.5 min) and final effect (10 min) the NLT had on hemodynamic variables for the four cohorts. Both a random intercept and random slope across time was specified for each participant. In this model, time (minutes) was a continuous variable and analysis of covariance (ANCOVA) was used to control for any differences between the baseline hemodynamic measurements among the four cohorts. The above models were controlled for age, sex, ethnicity, race, and BMI.

### Mediation analysis

The change in hemodynamic variables after completing the NLT were tested as potential mediators in the relationship between disease state and reduction in cognitive efficiency scores. Specifically, we used the change in PP and HR as the mediators and only looked at the immediate post-NLT change in cognitive efficiency scores. Our mediation models were examples of partial mediation. In our mediation analysis, we first considered the total effect between disease status and cognitive performance through simple linear regression. Second, again using simple linear regression, we evaluated the effect between disease status and orthostatic variables (HR and PP), which showed a significant effect. Third, we tested the effect of the orthostatic variables on cognitive performance. This was done using a regression model with both disease status and the change in HR/PP with the change in cognitive performance after the orthostatic challenge as our dependent variable. Lastly, we estimated causal mediation through bootstrapping (a nonparametric resampling method that allows for non-normal sampling distribution) using the mediation package in RStudio to compare the direct and indirect effects. The mediation models were adjusted or stratified for age, sex, time since diagnosis, and BMI due to their impact on the hemodynamic variables and cognition. We specifically considered 2 different models: (1) The mediating effect of change in HR and PP on the relationship between disease state and change in cognitive scores, when controlling for age, sex, and BMI and, (2) The mediating effect of change in HR and PP on the relationship between disease state and change in cognitive scores, when controlling for sex, BMI, and time since diagnosis, while additionally stratifying by age (<40 and ≥ 40). Less than 40 and ≥ 40 years was chosen because of similar distributions in each cohort (<40 group included 45 HCs, 15 PASC, 39 < 4 year ME/CFS and 23 > 10 year ME/CFS and the ≥40 group included 37 HCs, 19 PASC, 32 < 4 year ME/CFS and 42 > 10 year ME/CFS). All analyses were performed using RStudio (RStudio Team, 2020) with a significance level of *p* < 0.05.

### Missing and outlier data

Participants that were unable to finish the 10-min NLT or unable to complete cognitive testing after the NLT because of severe symptoms (e.g., fainting) were categorized as missing not at random. To account for missing hemodynamic data, we imputed the missing values by carrying forward the last recorded measurement. This method assumes their hemodynamic variables would not have changed if they had remained in the NLT and as a result, estimations are likely a lower bound on their individual treatment effect. To address the missing post-NLT cognitive measurements, we replaced the missing cognitive scores with the mean cognitive scores of those in the same cohort that had similar HR (+/− 5 bpm) at the time they stopped the NLT. Cognitive scores missing at baseline, 2 days and 7 days following the orthostatic challenge were assumed to be missing completely at random and were dropped from the analyses. Additionally, missing BMI, age, and sex measurements were assumed to be missing completely at random amongst the subjects and were also excluded from our final analyses. Outliers for cognitive scores were defined as being outside 4 standard deviations (SD) from the mean scores. There were 4 subjects with cognitive scores outside the −/+ 4 SD outlier thresholds. These outlier cognitive scores were replaced with cognitive scores minimum or maximum value at the +/− 4 SD threshold.

## Results

A total of 276 subjects were enrolled in the study (35 PASC, 75 < 4 year ME/CFS, 72 > 10 year ME/CFS, and 94 healthy controls). Twenty participants were excluded because they were missing their baseline DANA Brain Vital results or BMI measurements, resulting in 256 participants (34 PASC, 71 < 4 year ME/CFS, 69 > 10 year ME/CFS, and 82 healthy controls). Table 1 shows the demographics and clinical characteristics of the 256 participants included in the analyses.

**Table 1:** Cohort characteristics.

	PASC	ME/CFS		Healthy	*p* value
		<4 years	>10 years		
	*N* = 34	*N* = 71	*N* = 69	*N* = 82	
Age, years (±SD)	43 (±10.1)	39 (±13.2)	46 (±12.4)	40.0 (±13.3)	0.009
Sex					0.115
Female	29 (85%)	52 (73%)	50 (72%)	52 (63%)	
Male	5 (15%)	19 (27%)	19 (28%)	30 (37%)	
BMI	27.8 (±5.7)	25 (±5.4)	27.4 (±5.4)	26.3 (±5.3)	0.04
Ethnicity					0.1092
Hispanic	2 (6%)	4 (6%)	0 (0%)	1 (1%)	
Non-Hispanic	32 (94%)	65 (92%)	64 (93%)	78 (95%)	
Unknown	0 (0%)	1 (1%)	0 (0%)	0 (0%)	
Not reported	0 (0%)	1 (1%)	5 (7%)	3 (4%)	
Race					0.339
White	33 (97%)	69 (97%)	66 (96%)	77 (94%)	
Other	1 (3%)	0 (0%)	3 (4%)	2 (2%)	
Not reported	0 (0%)	2 (3%)	0 (0%)	3 (4%)	
Disease duration					
<1 year	28 (82%)	8 (11%)	0 (0%)	–	
<2 years	6 (18%)	21 (30%)	0 (0%)	–	
2–3 years	0 (0%)	25 (35%)	0 (0%)	–	
3–4 years	0 (0%)	17 (24%)	0 (0%)	–	
>10 years	0 (0%)	0 (0%)	69 (100%)	–	

Twenty-six (10.2%) participants were unable to finish the NLT and 11 (4.3%) participants were unable to take the DANA Brain Vital immediately after the NLT due to severe symptoms (e.g., fainting). Participants dropped out of the NLT throughout the 10-min testing period (Table 2) and dropout rates were similar among the 4 cohorts (3 PASC (8.8%), 7 < 4 year ME/CFS (9.9%), 9 > 10 year ME/CFS (13.0%), and 7 healthy controls (8.5%)).

**Table 2:** Number of participants that dropped out at a specific time during the NLT.

Minute of NLT	Number of participants that dropped out				Reason for dropping out
	PASC	<4 ME/CFS	>10 ME/CFS	HC	
1 min	0	0	0	0	
2 min	0	1	0	0	Fainted
3 min	0	0	0	3	Light headache, lightheaded, fainted
4 min	0	0	0	0	
5 min	0	0	0	2	Dizziness, fainted
6 min	0	0	0	2	Dizziness, fainted
7 min	2	2	0	0	Dizziness, fainted
8 min	1	2	4	0	Nausea, lightheaded, feeling faint
9 min	0	1	3	0	Fainted
10 min	0	1	2	0	Feeling shaky, lightheaded
Total	3	7	9	7	

**Figure 1:**
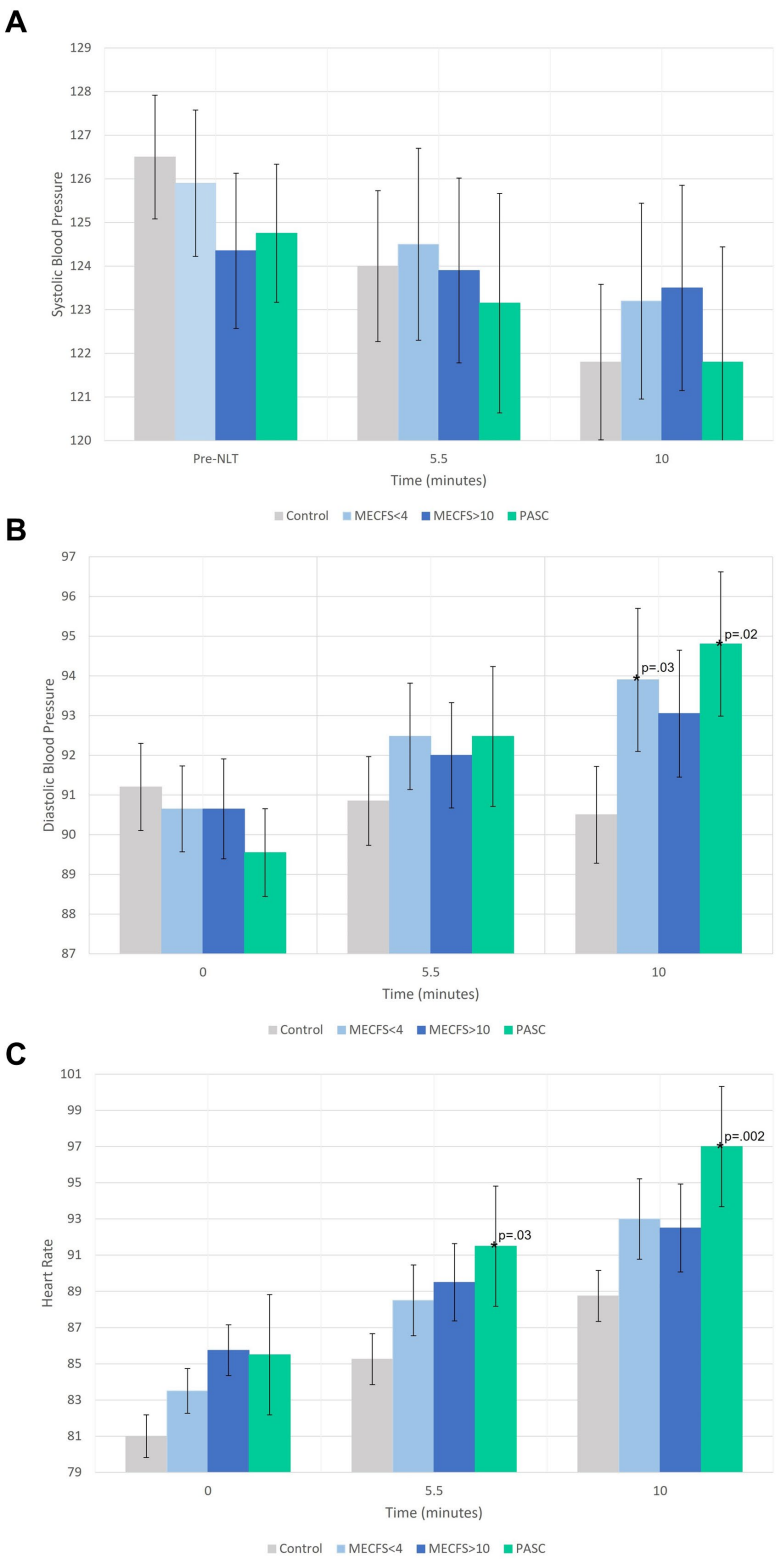
A comparison of the average effect at 5.5 min and the final effect at 10 min during the NLT on blood pressure and heart rate. In **(A)** SBP is within normal range for all cohorts. **(B)** Shows DBP increasing during the NLT for PASC, <4 year and > 10 year ME/CFS cohorts and decreasing during the NLT for the healthy control cohort. **(C)** Shows a significant HR increase for the PASC cohort while the ME/CFS cohorts increased but not significantly more than healthy controls.

The average effect at 5.5 min and the final effect at 10 min on hemodynamics during the NLT was examined first. There were no significant changes from baseline to 5.5 or 10 min for SBP for any of the disease cohorts compared to the healthy controls (Figure 1A). For both the <4 year ME/CFS and PASC cohorts, DBP was significantly higher than healthy controls at the end of the NLT (Figure 1B). The PASC cohort had significantly higher HR compared to healthy controls at both 5.5 min and at the end of the NLT (Figure 1C).

**Figure 2:**
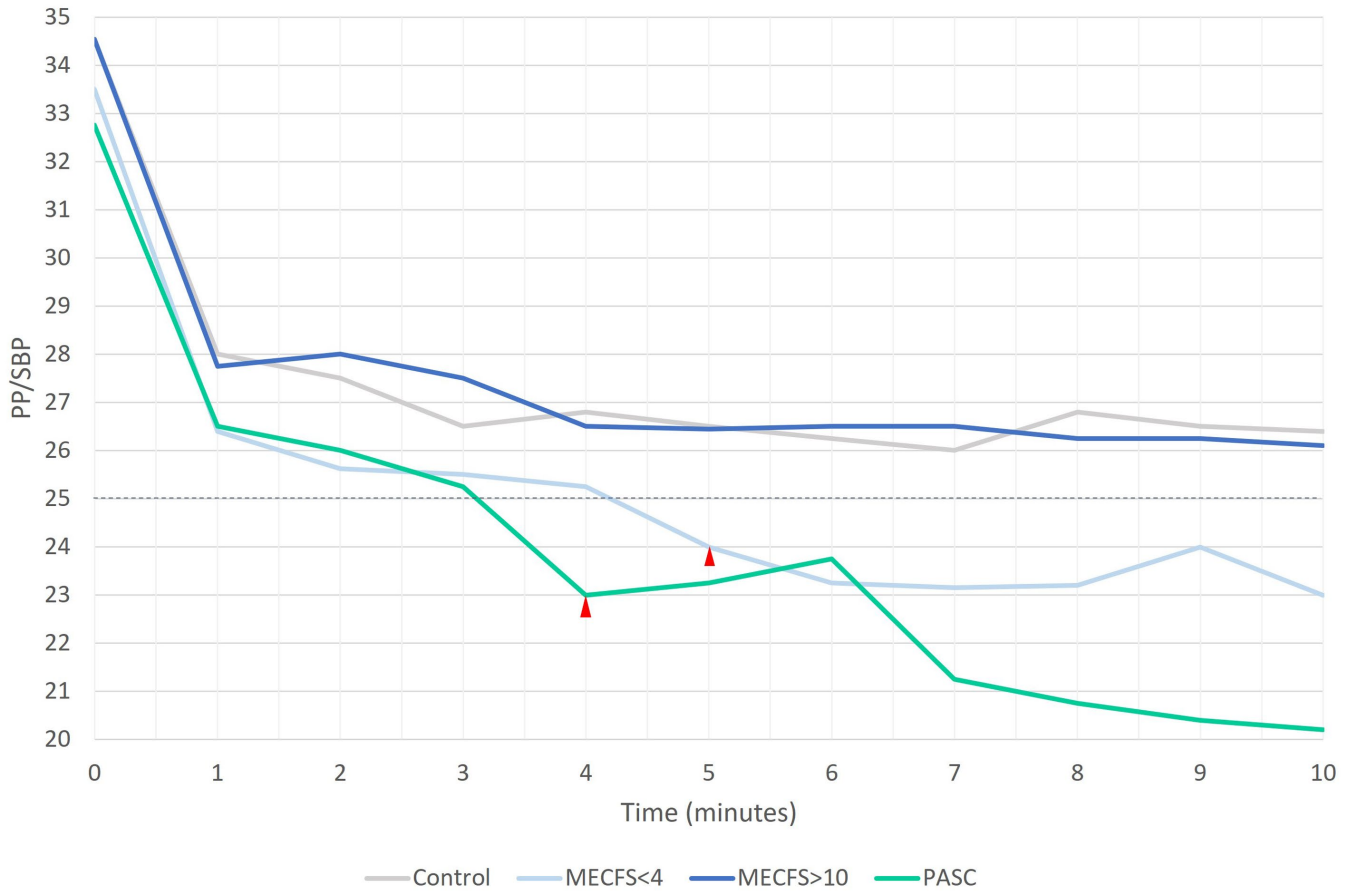
A PP that is less than 25% of SBP is inappropriately narrow. Both PASC and < 4 year ME/CFS cohorts have narrow PP by 4 and 5 min into the NLT, respectively.

When pulse pressure is less than 25% of SBP, PP is abnormally low or narrowed (Homan et al., 2022). Figure 2 shows the mean PP per minute of the NLT for each cohort. By 4 min into the NLT, the PP for the PASC and < 4 year ME/CFS cohorts narrowed to 23 and 24%, respectively. The PP continued to narrow with the PP dropping for the PASC cohort to 20 mmHg by the end of the NLT. The PP for the >10 year ME/CFS and healthy control cohorts were similar throughout the NLT and did not drop below 25% of SBP.

**Figure 3:**
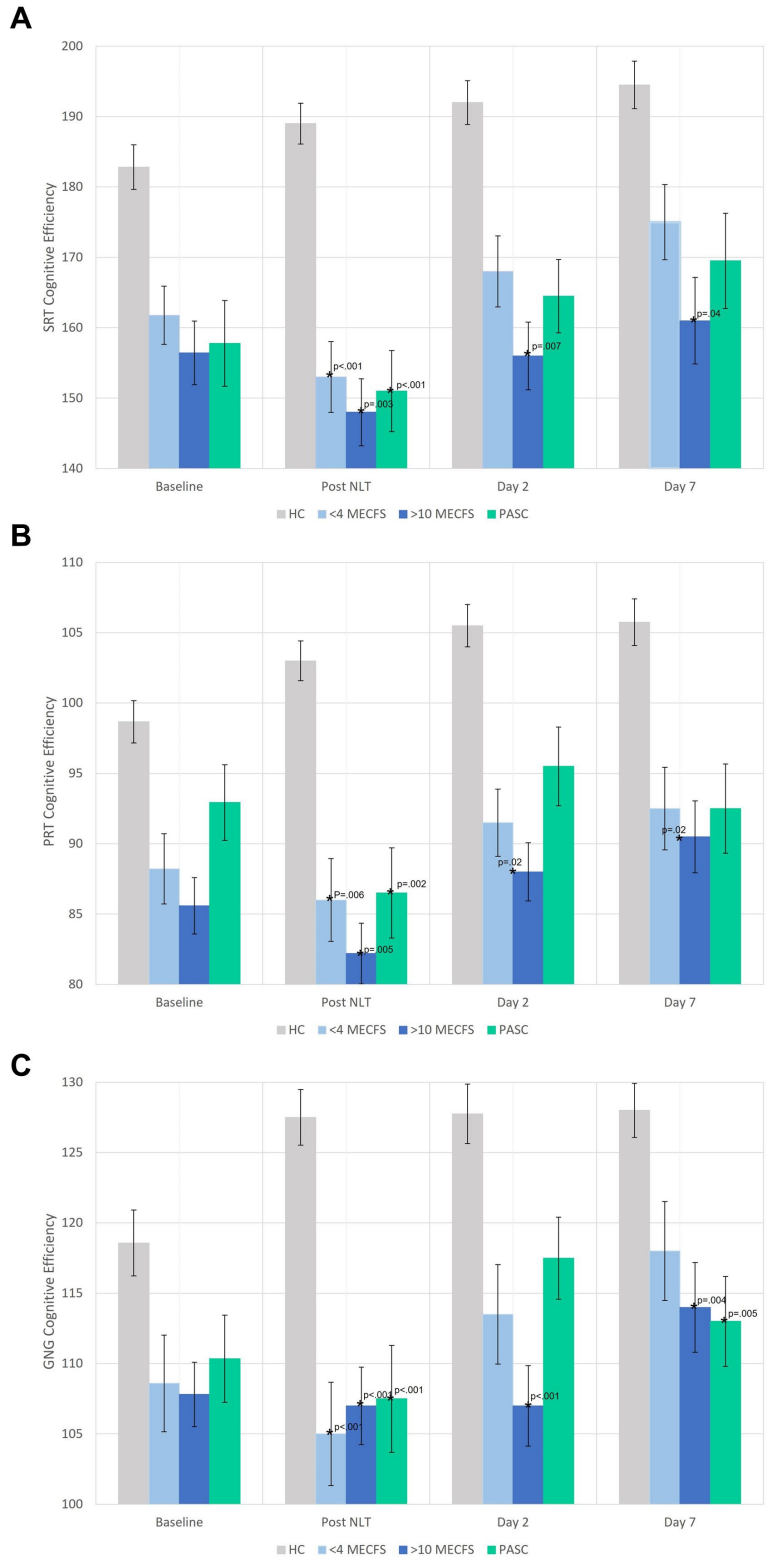
**(A–C)** DANA Brain Vital cognitive efficiency scores before and after the NLT. The disease cohorts had lower baseline cognitive efficiency scores on all three cognitive tests that significantly worsened immediately after the NLT. The >10 year ME/CFS cognitive efficiency scores remained significantly below healthy control cohort scores on days 2 and 7. The healthy control cohort had normal or improved cognitive efficiency scores after the NLT.

The cognitive efficiency scores before and after the NLT are shown in Figure 3. Notably, the disease cohorts had lower cognitive efficiency at baseline. The orthostatic challenge caused significant worsening of cognitive efficiency in all cognitive tests for the three disease cohorts compared to the healthy controls (Figures 3A–C). PASC and < 4 year ME/CFS participants returned to baseline cognitive efficiency levels by Day 2. On Day 7, GNG cognitive efficiency was significantly lower for PASC and > 10 year ME/CFS compared to <4 year ME/CFS and healthy controls (Figure 3C). Yet, the >10 year ME/CFS cohort had significantly lower cognitive efficiency scores compared to healthy controls at all time points for all three reaction time measures of the DANA Brain Vital battery (Figures 3A–C).

**Figure 4:**
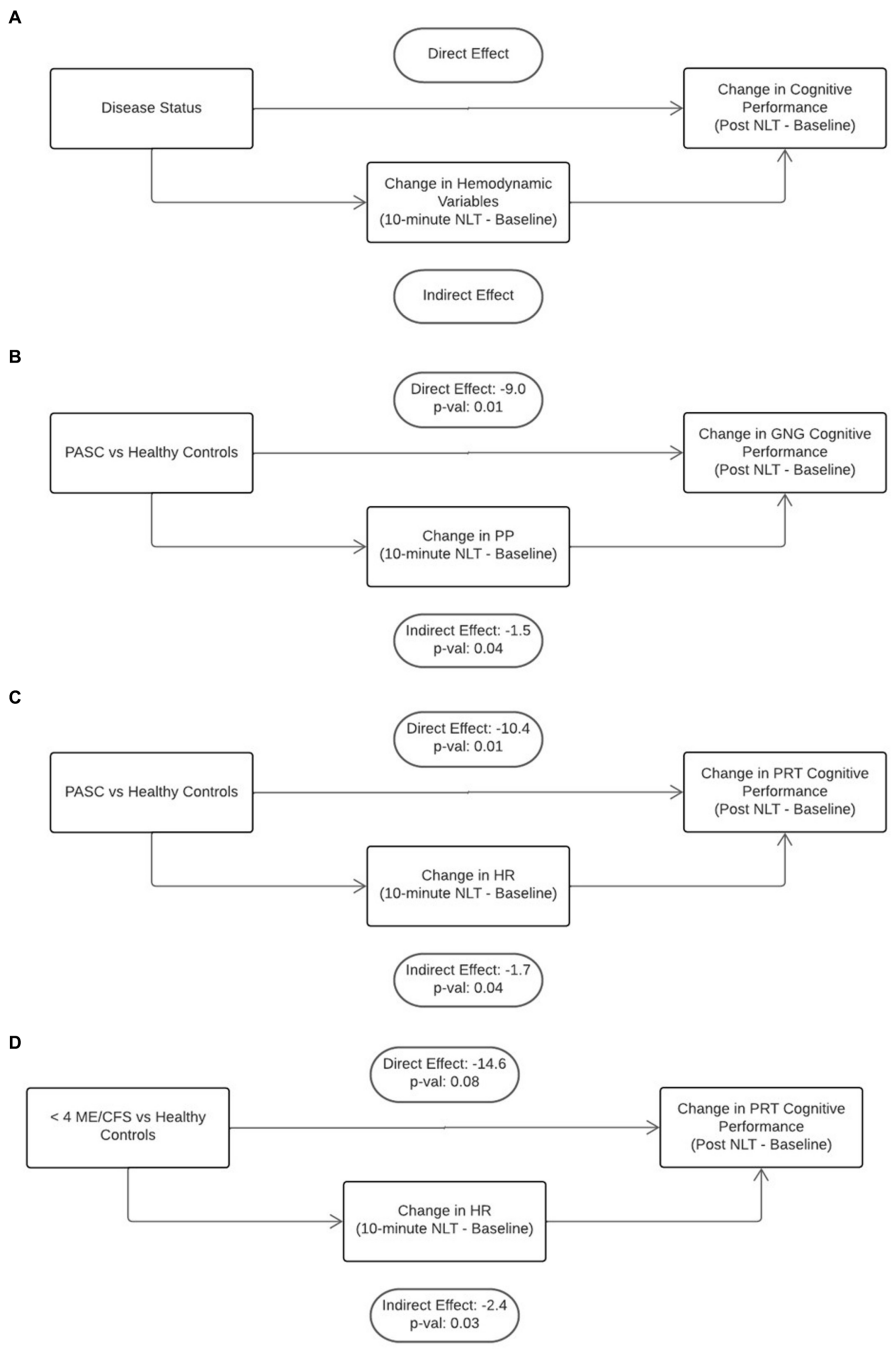
Mediation analysis was used to gain insight into the direct effect of disease on cognition and the indirect or mediating effect of orthostatic challenge on cognition **(A)**. There was a significant direct effect of PASC disease state as well as a significant indirect effect of decreased PP and increased HR on poorer cognitive efficiency **(B,C)**. The was a significant indirect effect of increased HR on worsened cognitive efficiency in the <4 year ME/CFS cohort **(D)**.

Mediation analysis was used to determine if the hemodynamic perturbations that occurred during the NLT were contributing to the decrease in cognitive efficiency (Figure 4A). In our first mediation model, when controlling for age, sex, and BMI, the change in PP in the PASC cohort during the NLT mediated slowed information processing assessed by GNG testing (−1.5, non-parametric bootstrap 95% CI: (−3.7, −0.04), *p* = 0.04) (Figure 4B). With change in PP as a mediator, there was a significant direct effect between the PASC vs. healthy control cohorts and change in cognitive performance (−9.0, non-parametric bootstrap 95% CI: (−15.8, −2.5), *p* = 0.01). (Figure 4B). Elevated HR that occurred during the NLT was not a significant mediator of slowed cognitive efficiency for any of the disease cohorts (data not shown).

The second mediation model stratified the cohorts into two age groups, <40 years and ≥ 40 years. This model found that a change in HR during the NLT was associated with slowed PRT in both the PASC (Figure 4C) and < 4 years ME/CFS cohorts (Figure 4D) in the ≥40 years group (PASC: −1.7, non-parametric bootstrap 95% CI: (−4.0.4, −0.04), p = 0.04 and ME/CFS <4 years: −2.4, non-parametric bootstrap 95% CI: (−6.2, −0.1), *p* = 0.03). There was a significant direct effect of PASC disease on slowed PRT (Figure 4C) (−10.4, non-parametric bootstrap 95% CI: (−18.0, −2.3), *p* = 0.01). The direct effect of <4 year ME/CFS disease on PRT did not reach significance (Figure 4D) (−14.6, non-parametric bootstrap 95% CI: (−27.5, 1.8), *p* = 0.08). Change in PP was not a significant mediator for disease state and cognitive efficiency for both age groups (data not shown).

## Discussion

There is significant clinical overlap of PASC with ME/CFS and because of this, research into the mechanisms of PASC may elucidate mechanisms of ME/CFS (Komaroff and Bateman, 2021; Komaroff and Lipkin, 2021; Choutka et al., 2022; Sukocheva et al., 2022). Recognizing these commonalities, several studies have documented dysautonomia and cognitive impairment in ME/CFS and PASC (Cook et al., 2017; Lee et al., 2020; Davis et al., 2021; Goodman et al., 2021; Wirth et al., 2021; Carmona-Torre et al., 2022; Gaglio et al., 2022; Natelson et al., 2022; Vernon et al., 2022; Walitt and Johnson, 2022; Jammoul et al., 2023). There is a consistent finding of a link between orthostatic intolerance and cerebral hypoperfusion causing corresponding cognitive deficits. This study is one of the first large scale, clinical research, time series analyses to compare short illness duration ME/CFS patients with PASC patients. Since OI is one of the common symptoms experienced by post-infection ME/CFS and PASC patients, we hypothesize that cerebral hypoperfusion that occurs from orthostatic challenge causes decreased cognitive efficiency and slower reaction times.

Orthostatic challenge is a test of venous return, atrial pre-load and ventricular filling. When a person moves from a lying to a standing position, blood pools in the lower extremities causing a drop in blood pressure that rapidly stabilizes in healthy people. PP is normal at 30–40 mmHg and is inappropriately narrow when it is 25% or less of systolic pressure. All cohorts showed an initial drop in PP when moving from lying to standing. Blood pressures stabilized for the remainder of the NLT showing no abnormal narrowing of pulse pressure for either healthy control or the >10 year ME/CFS cohorts. In contrast, PP progressively narrowed for both the PASC and < 4 year ME/CFS cohorts and by 4 and 5 min, PP was less than 25% of SBP. Abnormally narrowed pulse pressures occur in several diseases including heart failure (decreased pump effectiveness), blood loss, (decreased blood volume), aortic stenosis (reduced stroke volume), and cardiac tamponade (decreased filling time) and are due to decreases in systolic pressures while diastolic pressures remain stable (Homan et al., 2022). In contrast, we found that the narrow PP in PASC and < 4 year ME/CFS cohorts was due to a rise in DBP with relatively stable SBP. The mechanistic basis for the elevated DBP and narrowing pulse pressure during the orthostatic challenge is not clear for PASC but we hypothesize it may be a physiologically adaptive mechanism designed to mitigate the physiological stress of hemodynamic challenge. Research on ME/CFS patients indicates hemodynamic changes during orthostatic or exercise challenge results in reduced ventricular filling caused by the peripheral circulatory changes rather than primary cardiopulmonary perturbation (Lee et al., 2020; Singh et al., 2022).

There have been two reports of abnormally narrowed PP due to increased DBP in PASC and ME/CFS patients (van Campen and Visser, 2022; Vernon et al., 2022) but this is the first time narrow PP similar to PASC has been detected in short duration of illness ME/CFS patients. This points to the importance of illness duration studying ME/CFS and PASC. Forty one percent (29/71) of the <4 year ME/CFS cohort were sick for <2 years which makes these patients more comparable from a duration of illness perspective to the patients in the PASC cohort. The hemodynamic and cognitive similarities between the <4 year ME/CFS and PASC cohorts supports the post-viral commonalities and overlap between ME/CFS and PASC. The >10 year cohort experienced no significant hemodynamic changes during the NLT. We suspect that it is because these ME/CFS patients have been sick for a long time and experienced compensatory changes in heart rate in blood pressure response to chronic orthostatic stress even though cerebral perfusion remains abnormal (Shan et al., 2020). Nacul et al. proposed a framework for understanding the natural history of ME/CFS and made a strong case that the longer the disease duration, system abnormalities and cell dysfunction become more pronounced (Nacul et al., 2020).

All disease cohorts started with lower baseline cognitive efficiency prior to orthostatic stressor than the healthy controls and experienced further decrements in cognitive efficiency immediately after the NLT. DANA Brain Vital was designed to detect changes in cognitive function in response to trauma, illness, or exposure to environmental stressors (Lathan et al., 2013) and was effective in detecting the change in cognitive efficiency in response to the orthostatic challenge in this study as well. Slowed reaction time is one of the most sensitive measures of impaired cognitive functioning and is one of the cognitive domains with consistent evidence to be impaired in ME/CFS (Institute of Medicine, 2015; Coffman et al., 2018). Reduced cerebral blood flow due to hemodynamic decompensation during orthostatic challenge may explain why cognitive efficiency worsened immediately after the NLT (van Campen et al., 2020; Vernon et al., 2022). Both PASC and < 4 year ME/CFS cohorts returned to their baseline cognitive efficiency levels which were still below that of healthy controls.

The >10 year ME/CFS cohort had the lowest cognitive efficiency scores even though their hemodynamic response was similar to that of healthy controls. Cerebral perfusion is reduced in the ME/CFS patients and is not explained by a drop in blood pressure (van Campen et al., 2020). Chronic cerebral hypoperfusion is associated with neurocognitive disorders and cognitive impairment (Ciacciarelli et al., 2020) and it is possible that just being in an upright posture contributes to intermittent brain hypoperfusion and cognitive impairment. This could explain why the three disease cohorts all had low baseline cognitive efficiency scores; even coming to the clinic to participate in the study was an orthostatic stress. It is possible that the day 2 and 7 cognitive efficiency scores returned to baseline levels because testing was done at home.

Mediation analysis was used to determine the direct and indirect effects of disease state and orthostatic hemodynamic changes on cognitive impairment in PASC and ME/CFS. There was a significant direct effect of PASC on PRT and GNG cognitive efficiency. Neurological sequelae including cognitive impairment are known to occur following a variety of different viral infections (Clé et al., 2020). There was also a significant indirect effect of PP on GNG cognitive efficiency, implicating reduced cerebral blood flow on executive function. The GNG test targets executive functioning, recording speed and accuracy of targets, and omissions and commissions (Resnick and Lathan, 2016). Furthermore, increased HR in the PASC cohort had a significant indirect effect on PRT cognitive efficiency. The PRT test also targets executive functioning along with decision-making capabilities. Executive function includes processes such as working memory, attention, problem solving, and flexible thinking to successfully execute daily activities and goals. There is substantial evidence of neurologic involvement in acute COVID-19 and PASC (Hingorani et al., 2022) but this is one of the first studies to demonstrate the impact of orthostatic hemodynamic change on cognition in PASC.

Interestingly, there was a significant indirect effect of increased HR on lower PRT cognitive efficiency in the <4 year ME/CFS cohort. The direct effect of ME/CFS disease was not significant and notably, there were no significant effects in the mediation models for the >10 year ME/CFS cohort. This type of cognitive impairment is not explained by anoxic injury to the brain since the markers of tissue damage seen in other diseases characterized by chronic hypoperfusion have not been detected in ME/CFS (Singhal et al., 2002). It is possible that a cognitive impairment could be explained by oxidative stress from repeated ischemia–reperfusion injury that accompanies orthostatic intolerance and occurs from daily living with ME/CFS (Kell and Pretorius, 2022). This underscores the need for early diagnosis of PASC and ME/CFS to mitigate cognitive impairment and improve quality of life.

The predominant white female study population limits generalizing the results of this study to other nonwhite and other gender populations. The significant differences in age and BMI could affect the results of this study. However, that is not likely as the models controlled for sex, BMI, and time since diagnosis, while additionally stratifying by age (<40 and ≥ 40). The NLT is a point-of-care assessment that uses readily available equipment to monitor blood pressure and pulse oximetry that is easily implemented and analyzed in the clinic. However, the use of these methods is prone to user error and can give inaccurate readings as opposed to accurate and objective invasive blood pressure and heart rate monitoring. Since we used last observation carried forward to account for missing hemodynamic data for the most extreme cases, our estimates are likely an underestimate of the actual hemodynamic effects. The lack of deep hemodynamic data limits our ability to extrapolate these findings to specific pathways and pathophysiology.

In conclusion, being sick with PASC together with the accompanying orthostatic intolerance explained decreased cognitive efficiency that affected executive functioning. Poorer cognitive efficiency in ME/CFS patients sick for <4 years was partially associated with the likely adaptive response of elevated heart rate. It appears that the longer the duration of illness with ME/CFS, the more likely cognition is impaired in response to physiological stressors. Whether the cognitive deficits in executive function are stable, waxing-and-waning, or progressive is not known. Regardless, cognitive impairment affects daily life causing problems in remembering, concentrating and decision making. These findings underscore the need for early diagnosis and treatment of ME/CFS and PASC to improve daily cognitive functioning.

## Data availability statement

The raw data supporting the conclusions of this article will be made available by the authors, without undue reservation.

## Ethics statement

The studies involving human participants were reviewed and approved by The Jackson Laboratory Institutional Review Board (protocol 17-JGM-13). The patients/participants provided their written informed consent to participate in this study.

## Author contributions

SV, LB, and DU conceptualized the study design, supervised study implementation and assessments, and supervised the data collection. HD and GS were responsible for statistical analysis. SH, CR, BY, JB, and SA were responsible for patient recruitment and clinical assessment. SV and HD wrote the manuscript. SH, BY, CR, JB, SA, GS, DU, and LB reviewed and approved the manuscript. All authors contributed to the article and approved the submitted version.

## Funding

The study was funded by a grant to Derya Unutmaz from the National Institutes of Health (1U54NS105539).

## Conflict of interest

The authors declare that the research was conducted in the absence of any commercial or financial relationships that could be construed as a potential conflict of interest.

## Publisher’s note

All claims expressed in this article are solely those of the authors and do not necessarily represent those of their affiliated organizations, or those of the publisher, the editors and the reviewers. Any product that may be evaluated in this article, or claim that may be made by its manufacturer, is not guaranteed or endorsed by the publisher.

## Footnotes

^1^ https://covid19.who.int/

^2^ https://www.cdc.gov/nchs/pressroom/nchs_press_releases/2022/20220622.htm
